# Serum Derivatives–Reactive Oxygen Metabolite Levels as a Marker of Clinical Conditions in Patients with Bronchial Asthma, COPD, or Asthma–COPD Overlap: A Prospective Study

**DOI:** 10.3390/jcm13196022

**Published:** 2024-10-09

**Authors:** Keitaro Nakamoto, Masato Watanabe, Masaoki Saito, Keisuke Kasuga, Chika Miyaoka, Yuki Yoshida, Fumi Kobayashi, Hiroki Nunokawa, Jumpei Aso, Yasuo Nakamoto, Manabu Ishida, Mitsuru Sada, Kojiro Honda, Saori Takata, Takeshi Saraya, Masafumi Shimoda, Yoshiaki Tanaka, Mikio Saotome, Ken Ohta, Haruyuki Ishii

**Affiliations:** 1 Department of Respiratory Medicine, Kyorin University Faculty of Medicine, 6-20-2, Shinkawa, Mitaka-shi 181-8611, Tokyo, Japan; masato@ks.kyorin-u.ac.jp (M.W.); m-saito@ks.kyorin-u.ac.jp (M.S.); kkclub_0518@ks.kyorin-u.ac.jp (K.K.); chika@ks.kyorin-u.ac.jp (C.M.); yuki-yoshida@ks.kyorin-u.ac.jp (Y.Y.); kobayashi-fumi@ks.kyorin-u.ac.jp (F.K.); hrk910@ks.kyorin-u.ac.jp (H.N.); j_aso@ks.kyorin-u.ac.jp (J.A.); yasuo-nakamoto@ks.kyorin-u.ac.jp (Y.N.); matsu_manabu@ks.kyorin-u.ac.jp (M.I.); rainbow_orch@ks.kyorin-u.ac.jp (M.S.); h-kojiro78@ks.kyorin-u.ac.jp (K.H.); s-takata@ks.kyorin-u.ac.jp (S.T.); sarayatakeshi2@gmail.com (T.S.); h141@ks.kyorin-u.ac.jp (H.I.); 2 Department of Respiratory Medicine, Fukujuji Hospital, Japan Anti-Tuberculosis Association, 3-1-24, Matsuyama, Kiyose-shi 204-8522, Tokyo, Japan; shimodam@fukujuji.org (M.S.); tanakay@fukujuji.org (Y.T.); saotomem@fukujuji.org (M.S.); ohtak@fukujuji.org (K.O.)

**Keywords:** asthma–COPD overlap, bronchial asthma, COPD, oxidative stress, reactive oxygen metabolites

## Abstract

**Background:** Oxidative stress plays an important role in the pathophysiology of bronchial asthma (BA), chronic obstructive pulmonary disease (COPD), and asthma–COPD overlap (ACO), but its relevance has not been fully elucidated. The aim of this study was to measure the levels of oxidative stress and investigate its clinical significance in patients with BA, COPD, or ACO. **Methods:** We recruited 214 patients between June 2020 and May 2023 (109 patients with BA, 63 with COPD, and 42 with ACO). To assess clinical conditions, we evaluated patient characteristics, results of respiratory function tests and blood tests, and administered several questionnaires. We evaluated oxidative stress using the test for derivatives–reactive oxygen metabolites (d–ROMs) in serum. **Results:** The d–ROMs levels were significantly higher in patients with COPD or ACO than in patients with BA. There was no difference in serum d–ROMs levels between the COPD and ACO groups. In BA, d–ROMs levels were positively correlated with interleukin (IL)-6, IL-8, serum amyloid A (SAA), and C-reactive protein (CRP) levels; white blood cell (WBC) and neutrophil counts; and St. George’s Respiratory Questionnaire (SGRQ) scores, and they were negatively correlated with forced expiratory volume in 1 s (%FEV_1_) and asthma control test (ACT) score. In COPD, d–ROMs levels were positively correlated with IL-6, SAA, and CRP levels; WBC, neutrophil, and eosinophil counts; and COPD assessment test (CAT) and SGRQ scores, and they were negatively correlated with forced vital capacity (%FVC), %FEV_1_, and %FEV_1_/FVC scores. In ACO, d–ROMs levels were positively correlated with IL-6, SAA, tumor necrosis factor alpha (TNF-α), and CRP levels; and CAT and SGRQ scores, and they were negatively correlated with %FVC and %FEV_1_ scores. **Conclusions:** Serum d–ROMs levels may serve as a marker reflecting clinical conditions such as systemic inflammation, symptom severity, and airflow limitation in patients with BA, COPD, and ACO.

## 1. Introduction

Bronchial asthma (BA) and chronic obstructive pulmonary disease (COPD) are respiratory diseases characterized by chronic inflammation of the airways, resulting in airflow obstruction and restriction [1,2]. In BA, airway inflammation causes increased airway hyperresponsiveness and airway remodeling, resulting in symptoms such as cough, sputum production, shortness of breath, and wheezing. It can be exacerbated by a variety of factors, including respiratory infections, smoking, air pollution, allergen exposure, exercise, and stress [3,4]. In contrast, COPD causes chronic airway inflammation, resulting in difficulty in breathing, coughing, and sputum production, based on similar main risk factors such as smoking and air pollution [5,6]. The life prognosis of patients with COPD deteriorates due to exacerbations caused by respiratory infections and air pollution [7]. COPD is currently the third leading cause of death worldwide [8].

Although BA and COPD are different diseases, clinical practice frequently encounters patients who have both characteristics. For this reason, the Global Initiative for Asthma (GINA) and the Global Initiative for Chronic Obstructive Lung Disease (GOLD) jointly proposed the disease concept of asthma–COPD overlap (ACO). ACO has been defined as a disease characterized by chronic airflow obstruction that combines the characteristics of asthma and COPD [9].

While BA, COPD, and ACO have similar clinical features, it is often difficult to differentiate between the diseases, which can make selecting appropriate treatment difficult in some cases. Thus, useful markers reflecting clinical characteristics and disease status are needed.

Oxidative stress is known to occur when the balance between oxidative and antioxidative reactions in the body is disrupted. This can result in a state that causes oxidative damage to the body, and this phenomenon is involved in various diseases [10,11,12]. Reactive oxygen species (ROS) that cause oxidative stress include hydrogen peroxide, superoxide anions, hydroxyl radicals, and singlet oxygen. However, it is difficult to directly measure these, and no standardized clinical applications yet exist.

In recent years, a test for derivatives–reactive oxygen metabolites (d–ROMs) has become available that does not directly measure ROS but instead captures their metabolite ROOH and quantifies them as a measure of oxidative stress. Because of its ease of use, the test has been used in the study of various diseases, including respiratory diseases [13,14]. However, the relationship of oxidative stress to the pathophysiology of BA, COPD, and ACO has not yet been elucidated. In the present study, therefore, we measured serum d–ROMs levels as an oxidative stress marker and investigated their relationship to the clinical conditions of BA, COPD, and ACO. We primarily aimed to determine serum d–ROMs levels differences between BA, COPD and ACO. The secondary aim was to determine the correlation between serum d–ROMs levels and laboratory data, pulmonary function tests, and respiratory questionnaire scores in each disease.

## 2. Materials and Methods

### 2.1. Patients and Study Design

This study was designed as a prospective cohort study. We prospectively recruited patients with BA, COPD, or ACO who visited Kyorin University or Fukujuji Hospital from June 2020 until May 2023. Recruited patients with BA and COPD fulfilled the definition criteria of GINA and GOLD, respectively [1,2]. We excluded patients under 18 years of age and those who had experienced any exacerbation within four weeks prior to the study enrollment. All patients provided written informed consent prior to participation in this study. The institutional review boards at Kyorin University and Fukujuji Hospital approved this study (approvals no. 755 and no. 20026, respectively).

Patients were diagnosed with ACO when they fulfilled the criteria of the Japanese Respiratory Society (JRS) (Table 1) [15]. These criteria comprise being older than 40 years of age, having a value for post-bronchodilator forced expiratory volume in 1 s (FEV_1_) divided by forced vital capacity (FVC) of <0.7, and fulfilling at least one of the following criteria: a smoking history of more than 10 pack-years, the presence of a low attenuation area (LAA) on high-resolution computed tomography (HRCT), or a value for diffusing capacity of the lung for CO (D_LCO_) divided by alveolar volume (VA) of <0.8. In addition, ACO was diagnosed if patients with COPD fulfilled at least two of the following criteria: variable (diurnal, daily, or seasonal) or paroxysmal respiratory symptoms (cough, sputum, or dyspnea), an asthma history prior to reaching 40 years of age, or fractional exhaled nitric oxide (FeNO) > 35 ppb; or if they fulfilled one of these criteria and at least two of the following criteria: perennial allergic rhinitis, airway reversibility (change in FEV_1_ > 12% and >200 mL), blood eosinophil count >5% or >300 cells/μL, or elevated total immunoglobulin E (IgE), or positive specific IgE antibody.

### 2.2. Laboratory Data

At the time of enrollment, all patients were ordinary tested for white blood cell (WBC), neutrophil count, eosinophil count, C-reactive protein (CRP), serum amyloid A (SAA), IgE and the following specific IgE antibodies: house dust mite (*Dermatophagoides pteronyssinus*), cedar pollen, cypress pollen, alder pollen, orchard grass pollen, ragweed pollen, mugwort pollen, cat, dog, fungi (*Alternaria*, *Aspergillus*), moth, chironomid and cockroach.

### 2.3. Measurement of Serum IL-6, IL-8 and TNF-α

Serum interleukin (IL)-6, IL-8 and tumor necrosis factor alpha (TNF-α) were measured using an ELISA kit (R&D Systems, Minneapolis, MN, USA).

### 2.4. Measurement of Serum d–ROMs Levels

To assess serum oxidative stress, we performed d–ROMs tests using the FREE Carpe Diem system (Diacron International, Grosseto, Italy) according to the manufacturer’s instructions. Briefly, a serum sample (20 μL) was placed in a cuvette filled with acid buffer. Iron ions served as catalysts, resulting in the degradation of hydroperoxide into free radicals (alkoxyl and hydroperoxyl radicals). When the colorless oxidizable chromogenic mixture was added to this solution in a volume of 20 μL, this resulted in oxidation of the chromogen substrate by the free radicals to yield a red-colored radical cation. The final solution can be observed by the absorbance change per minute at 505 nm. Levels are expressed in units of CARR U, equivalent to 0.08 mg/dL of hydrogen peroxide. The normal range of this test was established as 200–300 CARR U, with borderline, low, middle, and high levels defined as 301–320, 321–340, 341–400, and >400 CARR U, respectively [16].

### 2.5. Pulmonary Function Tests and Fractional Exhaled Nitric Oxide (FeNO) Level

Pulmonary function tests were performed using a SYSTEM 21 device (MINATO MEDICAL SCIENCE, Osaka, Japan) or CHESTSTAC8800 device (Chest M.I., Tokyo, Japan) according to the criteria of the American Thoracic Society (ATS), the European Respiratory Society (ERS), and the Japanese Respiratory Society (JRS). The FeNO level was measured using a NIOX VERO device (Aerocrine, Solna, Sweden) or NO breath device (Bedfont Scientific, Maidstone, UK) according to the manufacturer’s instructions and the ATS guidelines.

### 2.6. Respiratory Questionnaire Scores

The patient’s condition was evaluated using asthma control test (ACT) [17], COPD assessment test (CAT) [18] and St. George’s Respiratory Questionnaire (SGRQ) scores [19] in this study.

### 2.7. Statistical Analysis

All data were expressed as median (interquartile range) or percentages (%). Statistical analyses were carried out using GraphPad Prism 10.1.2 (GraphPad Software, La Jolla, CA, USA). Comparisons between quantitative variables for two groups were conducted using the Mann–Whitney test, and those between quantitative variables for three groups were conducted using the Kruskal–Wallis test, followed by Dunn’s *post hoc* test. Within-group differences between qualitative variables were evaluated using the Chi-square test. Spearman’s rank test was used to assess correlations among variables. A *p*-value < 0.05 was considered to indicate statistical significance. Receiver operating characteristic (ROC) curves were used to assess the discriminative power of serum d–ROMs levels. The cutoff point for serum d–ROMs levels was determined using Youden’s index.

## 3. Results

### 3.1. Patient Characteristics

A total of 214 patients were enrolled in this study, of whom 109 were diagnosed with BA (median age 64 years; 44 were men), 63 were diagnosed with COPD (median age 78 years; 53 were men), and 42 were diagnosed with ACO (median age 73 years; 36 were men) (Table 2). The frequency of inhaled corticosteroids (ICS) use was higher in the BA (93.6%) and ACO groups (81.0%) than in the COPD group (25.4%) (*p* < 0.001, *p* < 0.001, respectively). The frequency of long-acting muscarinic antagonist (LAMA) use was higher in the COPD (73.0%) and ACO groups (64.3%) than in the BA group (21.1%) (*p* < 0.001, *p* < 0.001, respectively). There were no differences in the frequency of long-acting beta2-agonist (LABA) use among the three patient groups. The frequency of oral corticosteroids (OCS) and biologics use were higher in the BA group (12,8%, 12.8%) than in the COPD (0%, 0%) and ACO groups (9.5%, 7.1%) (*p* < 0.003, *p* < 0.003, *p* < 0.012, *p* < 0.031, respectively).

### 3.2. Serum d–ROMs Levels

**Figure 1:**
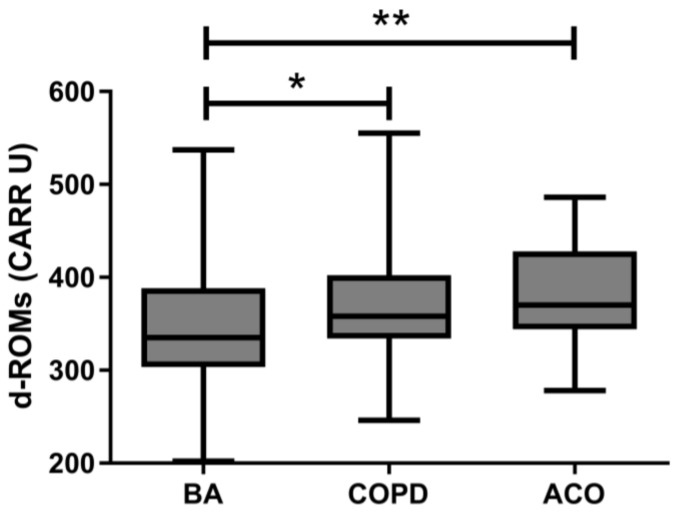
Serum d–ROMs levels in patients with BA, COPD, or ACO. d–ROMs, derivatives–reactive oxygen metabolites; BA, bronchial asthma; COPD, chronic obstructive pulmonary disease; ACO, asthma–COPD overlap; * *p* < 0.05, ** *p* < 0.01.

**Figure 2:**
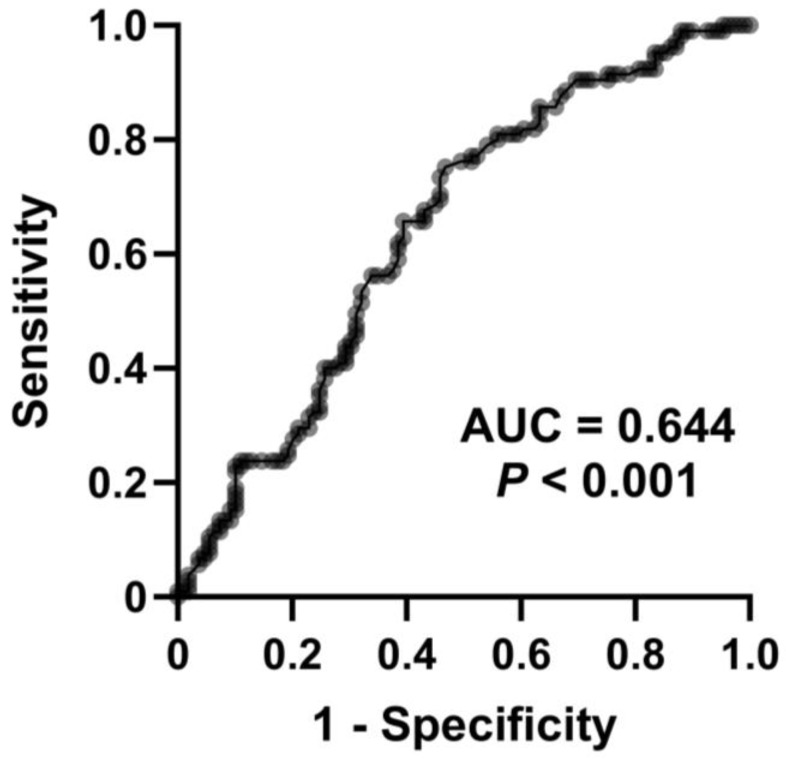
Receiver operating characteristic curve for serum d–ROMs levels to discriminate BA from COPD and ACO. AUC, area under the curve.

The median serum d–ROMs level of all patients enrolled in this study was 352 (313–400) CARR U. Median levels were significantly higher in the COPD and ACO groups than the BA group (358 CARR U vs. 335 CARR U, *p* = 0.018; 370 CARR U vs. 335 CARR U, *p* = 0.004, respectively) (Figure 1). There was no difference in serum d–ROMs levels between the COPD and ACO groups. The cutoff value for discriminating BA from COPD/ACO was calculated as 337 CARR U (sensitivity 75.2%, specificity 53.2%, positive predictive value 60.8%, negative predictive value 69.0%). The area under the curve (AUC) in the ROC analysis was 0.644 (95% confidence interval: 0.570–0.718, *p* < 0.001) (Figure 2). Serum d–ROMs levels are thus elevated in the presence of COPD.

### 3.3. Relationship between Serum d–ROMs Levels and Biomarkers

In the BA group, serum d–ROMs levels were correlated with IL-6, IL-8, SAA, and CRP levels and with white blood cell (WBC) and neutrophil counts (*rs* = 0270, *p* = 0.004; *rs* = 0.232, *p* = 0.015; *rs* = 0.316, *p* = 0.001; *rs* = 0.375, *p* < 0.001; *rs* = 0.253, *p* = 0.008; *rs* = 0.293, *p* = 0.002, respectively) (Table 3). In the COPD group, serum d–ROMs levels were correlated with IL-6, SAA, and CRP levels and with WBC, neutrophil, and eosinophil counts (*rs* = 0487, *p* < 0.001; *rs* = 0.408, *p* < 0.001; *rs* = 0.431, *p* < 0.001; *rs* = 0.265, *p* = 0.036; *rs* = 0.265, *p* = 0.036; *rs* = 0.431, *p* < 0.001, respectively). In the ACO group, serum d–ROMs levels were correlated with IL-6, SAA, TNF-α, and CRP levels (*rs* = 0483, *p* = 0.001; *rs* = 0.487, *p* = 0.001; *rs* = 0.417, *p* = 0.006; *rs* = 0.440, *p* = 0.004, respectively). Serum d–ROMs levels were not correlated with IgE in any patient group. In summary, serum d–ROMs levels were correlated with systemic inflammation markers in all three diseases.

### 3.4. Relationship between Serum d–ROMs Levels and Pulmonary Function Test

**Figure 3:**
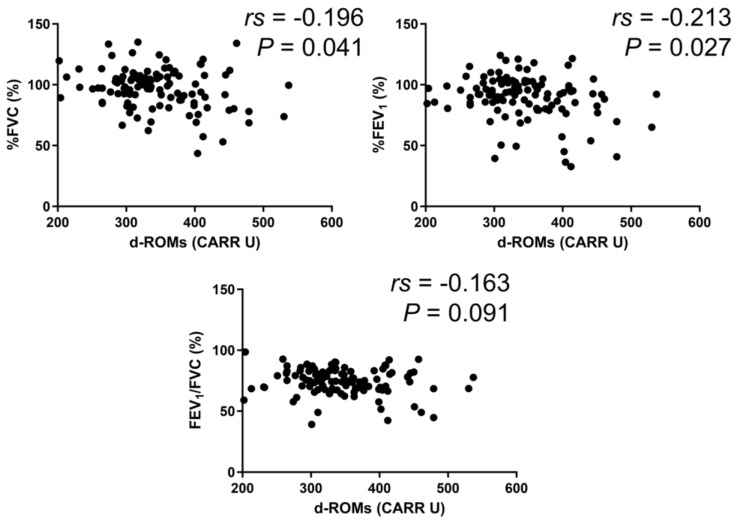
Correlation between serum d–ROMs levels and lung function test parameters in BA. FVC, forced vital capacity; FEV1, forced expiratory volume in 1 s.

**Figure 4:**
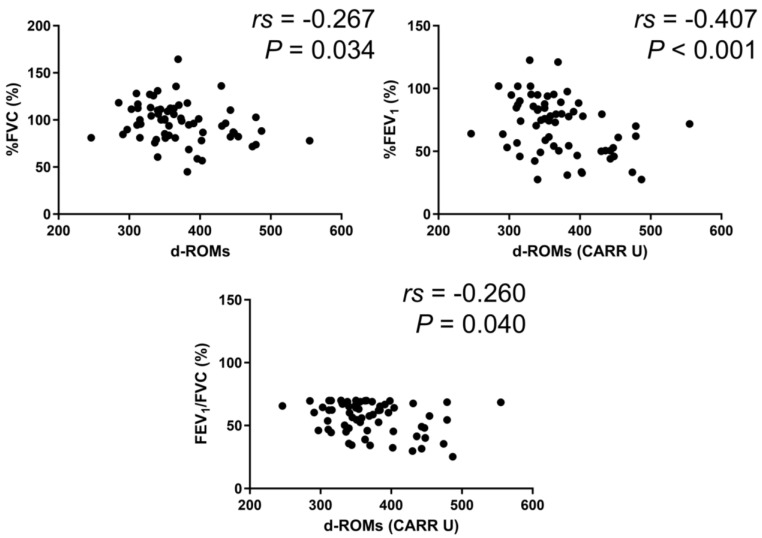
Correlation between serum d–ROMs levels and lung function test parameters in COPD.

**Figure 5:**
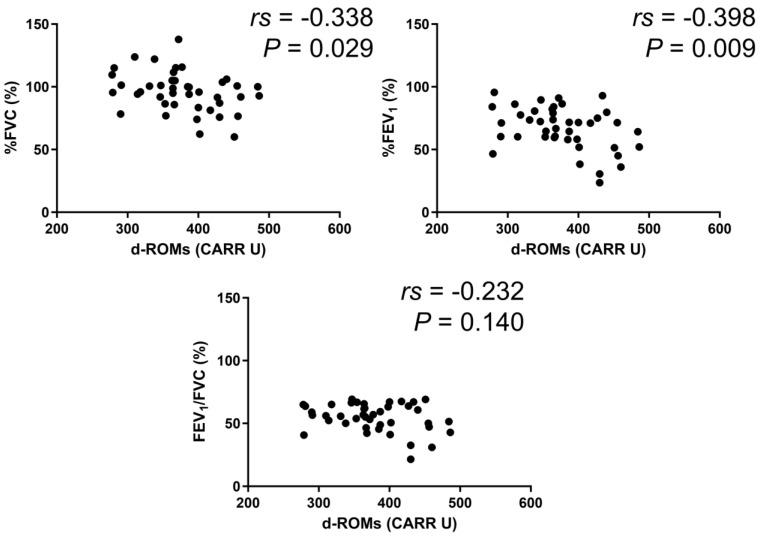
Correlation between serum d–ROMs levels and lung function test parameters in ACO.

In the BA group, serum d–ROMs levels were negatively correlated with %FEV_1_ (*rs* = −0.213, *p* = 0.027) (Figure 3). In the COPD group, serum d–ROMs levels were negatively correlated with %FVC, %FEV_1_, and %FEV_1_/FVC (*rs* = −0.267, *p* = 0.034; *rs* = −0.407, *p* < 0.001; *rs* = −0.260, *p* = 0.040, respectively) (Figure 4). In the ACO group, serum d–ROMs levels were negatively correlated with %FVC and %FEV_1_ (*rs* = −0.338, *p* = 0.029; *rs* = −0.398, *p* = 0.009, respectively) (Figure 5). Serum d–ROMs levels were not correlated with FeNO in any group (Table 3). These findings indicate that serum d–ROMs levels can reflect the degree of airflow limitation in these diseases.

### 3.5. Relationship between Serum d–ROMs Levels and Respiratory Questionnaire Scores

**Figure 6:**
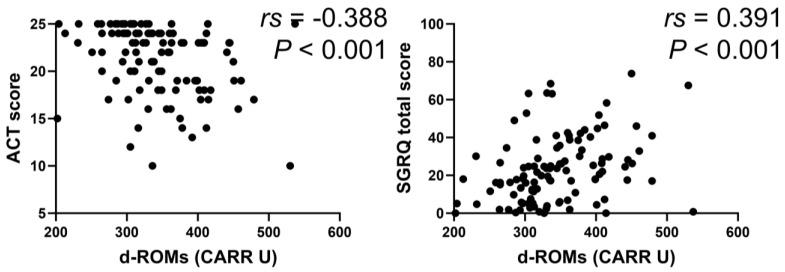
Correlation between serum d–ROMs levels and respiratory questionnaire score in BA. ACT, asthma control test; SGRQ, St. George’s Respiratory Questionnaire.

**Figure 7:**
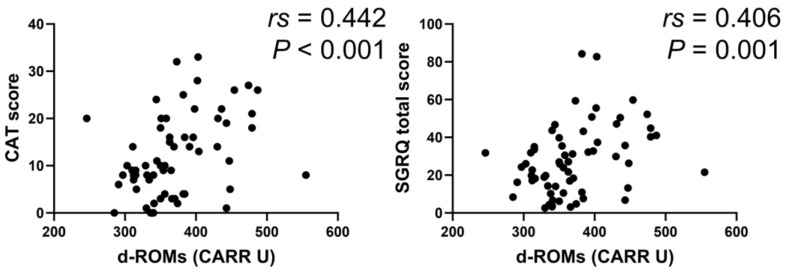
Correlation between serum d–ROMs levels and respiratory questionnaire score in COPD. CAT, COPD assessment test.

**Figure 8:**
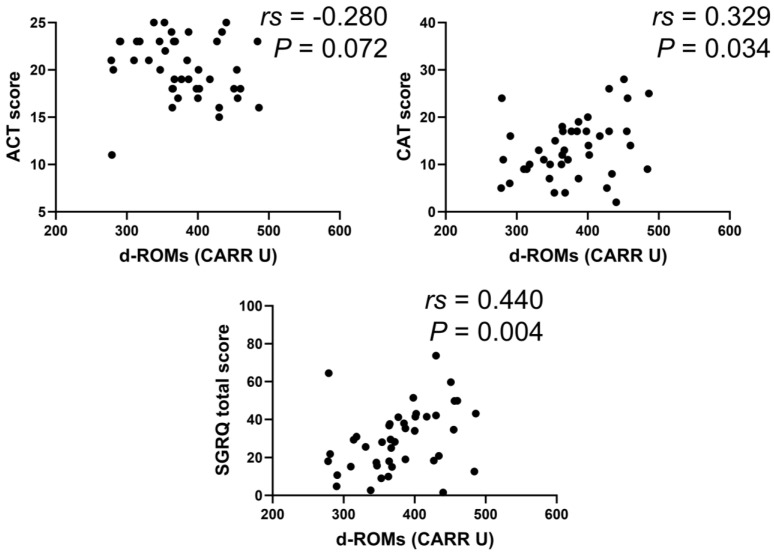
Correlation between serum d–ROMs levels and respiratory questionnaire score in ACO.

In the BA group, serum d–ROMs levels were negatively correlated with ACT score (*rs* = −0.388, *p* < 0.001) and positively with total SGRQ score (*rs* = 0.391, *p* < 0.001) (Figure 6). In the COPD group, serum d–ROMs levels were positively correlated with CAT and total SGRQ scores (*rs* = 0.442, *p* < 0.001; *rs* = 0.406, *p* = 0.001, respectively) (Figure 7); this was also the case in the ACO group (*rs* = 0.329, *p* = 0.034, *rs* = 0.440, *p* < 0.004, respectively) (Figure 8).

With respect to the SGRQ score, all components of symptom, activity, and impacts were correlated with serum d–ROMs levels in the BA (*rs* = 0.282, *p* = 0.003; *rs* = 0.390, *p* < 0.001; *rs* = 0.297, *p* = 0.002, respectively) and COPD (*rs* = 0.382, *p* = 0.002; *rs* = 0.286, *p* = 0.023; *rs* = 0.411, *p* = 0.001, respectively) groups. In the ACO group, the activity component was correlated with serum d–ROMs levels (*rs* = 0.440, *p* = 0.004), but no correlation was found with the symptom and impacts components (*rs* = 0.304, *p* = 0.050; *rs* = 0.286, *p* = 0.066, respectively). In summary, serum d–ROMs levels reflect the severity of symptoms in these diseases.

## 4. Discussion

In this study, we evaluated the serum d–ROMs levels of patients with BA, COPD, or ACO to assess oxidative stress. We found that they were higher in patients with COPD or ACO than in those with BA. We also found that higher serum d–ROMs levels were associated with greater systemic inflammation, symptom severity, and airflow limitation in patients with any of these conditions.

Various recent studies have reported that oxidative stress is associated with the pathophysiology of BA [20,21,22]. For instance, Kotsiou et al. reported that both d–ROMs and the plasma antioxidant capacity test showed high levels of oxidative stress in severe asthmatics with controlled asthma [23]. Furthermore, In a previous study that also focused on the relationship between oxidative stress and BA, we reported that patients with high serum d–ROMs levels were more likely to experience exacerbation within three months of measurement than those with low levels [24]. We also previously reported that serum d–ROM levels were positively correlated with levels of IL-6 and CRP and negatively correlated with %FEV_1_. In contrast, in the present study, serum d–ROMs levels in patients with BA were positively correlated with serum levels of WBC, CRP, SAA, IL-6, and IL-8 and negatively correlated with %FEV_1_. These two studies show that oxidative stress in patients with BA is associated with disease severity, systemic inflammation, and airflow limitation.

In addition, the present study showed that serum d–ROMs levels were significantly correlated with symptom scores. Symptom scores have been reported to be related to BA severity and used in several studies [25,26,27]. Although the ACT is a very simple test, its scores successfully discriminated between groups of patients who had received differing specialist ratings of asthma control and percent predicted FEV_1_ [25]. The SGRQ total score is also considered a very useful measure, and its results have similarly been reported to be associated with BA [26]. Although questionnaires have the benefit of being noninvasive, they can sometimes be cumbersome and time-consuming to administer. They may also be difficult to use with patients with reduced cognitive function because they are subjective measures. In comparison, the d–ROMs test is based on blood samples and therefore has advantages in terms of objectivity and reproducibility. Since only a small amount of blood (20 μL) is required and the measurement can be carried out in a short period of time, it may also hold promise as a new testing tool for evaluating medical conditions. Of note, the d–ROMs test can be applied for any patients to determine the disease status soon after the time of sample collection. The results of our study suggest that d–ROMs measurement could be a useful rapid test for BA management.

Smoking is one of the main causes of COPD and a major cause of oxidative stress in the lungs [28,29,30]. Since chronic exposure to oxidative stress and persistent inflammation can lead to emphysema, oxidative stress and COPD are closely related. As was reported in the asthma group, various biomarkers of oxidative stress including the d–ROMs test have previously been reported [31]. Melillo et al. reported that d–ROMs levels are higher in patients with COPD than healthy individuals [32]. Yamamura et al. previously reported that d–ROMs levels are associated with COPD severity and airway obstruction [13]. In the present study, we similarly found that serum d–ROMs levels were negatively correlated with %FVC, %FEV_1_, and FEV_1_/FVC. Further, we also found positive correlations with inflammatory markers such as IL-6 levels and symptom scores (CAT score and SGRQ total score). COPD is also considered a systemic inflammatory disease, and serum IL-6 and TNF-α have been reported as markers that reflect the condition’s severity [33]. It has been reported that the CAT score, a metric that evaluates the degree of airflow obstruction in COPD, is also correlated with %FEV_1_ [34]. Therefore, the present results suggest that serum d–ROMs levels represent a useful marker that reflects disease severity, systemic inflammation, and airflow limitation in both COPD and BA.

As described by the GOLD and GINA initiatives, ACO is a disease that combines features of both BA and COPD. Although some characteristics of this disease have been delineated, no clear diagnostic criteria have yet been provided [1,2]. Since the diagnosis guidelines of the JRS are objective and clear due to their use biomarkers and specific numerical values, we used them in the present study to diagnose patients with ACO [15]. These criteria are determined by separately examining items relating to BA and COPD after an obstructive disorder occurs, and they are thus relatively easy to use. Similarly to the results for BA and COPD, the present study showed that serum oxidative stress in patients with ACO was associated with inflammatory markers, pulmonary function, and symptom scores. In other words, serum d–ROMs levels were positively correlated with CAT scores, SGRQ total scores, and CRP, IL-6, and SAA levels, and negatively correlated with %FVC and %FEV_1_. To the best of our knowledge, this is the first report showing a relationship between ACO and oxidative stress, and our findings underline the applicability of measuring oxidative stress in ACO diagnosis.

The characteristics of ACO have been reported in several studies. For instance, Miravitlles et al. reported that ACO patients were more likely to display symptoms such as dyspnea and wheezing [35]. They also reported observing no differences in systemic inflammatory markers such as IL-6, IL-8, and CRP between ACO and COPD. Menezes et al. reported that ACO was associated with higher risks for exacerbations and hospitalizations than COPD [36]. In the present study, we found no differences in age, gender ratio, or systemic inflammatory markers (IL-6, IL-8, SAA, TNF-α, and CRP) between the COPD and ACO groups, similar to the findings of a previous report [35]. Further, we also found that serum d–ROMs levels in patients with ACO were significantly higher than in those with BA but did not significantly differ from those with COPD. These results may reflect the absence of significant differences in age or gender ratio between the COPD and ACO groups. In addition, since ACO shares some of the characteristics of COPD, it is possible that oxidative stress in the ACO group was higher than in the BA group because patients were more heavily affected by systemic inflammation, which is a characteristic of COPD. Although interpretation of the ROC curve (Figure 2), which represents a preliminary finding, does not allow a firm conclusion, it is possible that measurement of oxidative stress may serve as an auxiliary tool for differentiating BA from COPD and ACO. Moreover, we found that serum d–ROMs levels were not only negatively correlated with pulmonary function but also associated with symptom scores and systemic inflammatory markers. We therefore suggest that oxidative stress could be a useful test for evaluating the pathology of ACO, similarly to BA and COPD.

Based on our findings, high serum d–ROMs levels (>337 CARR U) indicate the presence of COPD, with a sensitivity of 75.2%. It is interesting to consider that the serum d–ROMs test may serve as a clinical indicator for COPD and contribute to precise diagnosis and optimization of treatment. Further research is required to clarify this possibility.

This study is subject to several limitations. First, it was a relatively small-scale study, so our findings may require further confirmation by larger-scale studies. Second, we included no healthy controls because this would have interfered with the use of questionnaires and respiratory function tests. Third, we enrolled moderately well controlled patients after exclusion of those who had recent exacerbation prior to enrollment. Therefore, we cannot determine the effect of the exacerbation on the d–ROMs levels. Further, we evaluated serum oxidative stress markers, but not airway substances such as exhaled breath condensate. Unfortunately, we did not examine other oxidative stress biomarkers such as H_2_O_2_, malondialdehyde, 8-isoprostane, vitamin C, and vitamin E and their correlation with the d–ROMs test.

Finally, the two hospitals participating in the study used different equipment for administering pulmonary function tests and FeNO analysis, which may have affected the results slightly. Nevertheless, our study shows that d–ROMs can be used as a marker to understand the clinical conditions of BA, COPD, and ACO, which supersedes the aforementioned limitations.

## 5. Conclusions

In this study, we evaluated the use of oxidative stress indicators as markers of obstructive pulmonary diseases such as BA, COPD, and ACO using the d–ROMs test. We found that serum d–ROMs levels were higher in patients with COPD or ACO than in those with BA, and that oxidative stress in each disease was associated with inflammatory markers, pulmonary function, and symptom scores. Oxidative stress may serve as a marker reflecting clinical conditions such as systemic inflammation, symptoms, and airflow limitation in these diseases.

## Figures and Tables

**Table 1:** JRS diagnostic criteria for ACO.

Basic Characteristics	
Post-bronchodilator FEV_1_/FVC < 70% in individuals 40 years of age or older	
Exclusion of other pulmonary diseases by chest X-ray and other relevant techniques	
**Features of BA**	**Features of COPD**
2 items from 1–3 apply or 1 item from 1–3 and 2 items from 4 apply.	1 item from 1–3 apply.
1. Variable (diurnally, daily, seasonally) or paroxysmal respiratory symptoms (cough, sputum, dyspnea)	1. Smoking history (≥10 pack-years) or same level of air pollution exposure
2. BA diagnosis history under 40 years	2. Presence of low attenuation area showing emphysematous changes on chest CT
3. FeNO > 35 ppb	3. Impaired pulmonary diffusing capacity (D_LCO_ < 0.8 or D_LCO_/V_A_ < 0.8)
4-1. Complication of allergic rhinitis	
4-2. Bronchodilator response of FEV_1_ ≥ 200 mL and 12% from baseline	
4-3. Peripheral blood eosinophil count > 5% or 300 cells/μL	
4-4. High levels of IgE (total IgE or specific IgE against perennial inhalant antigens)	

JRS, the Japanese Respiratory Society; ACO, asthma–COPD overlap; BA, bronchial asthma; COPD, chronic obstructive pulmonary disease; FEV_1_, forced expiratory volume in 1 s; FVC, forced vital capacity; D_LCO_, diffusing capacity of the lung for carbon monoxide; FeNO, fractional exhaled nitric oxide; IgE, immunoglobulin E.

**Table 2:** Patient characteristics.

	BA	COPD	ACO	*p*	*p*	*p*
	N = 109	N = 63	N = 42	BA vs. COPD	BA vs. ACO	COPD vs. ACO
Age, years	64 (51–74)	78 (73–83)	73 (66–79)	<0.001	0.001	0.058
Gender, male	44 (40.4)	53 (84.1)	36 (85.7)	<0.001	<0.001	0.825
Never smoker	71 (65.1)	0 (0.0)	0 (0.0)	<0.001	<0.001	1.000
Ex smoker	33 (30.3)	50 (79.4)	35 (83.3)	<0.001	<0.001	0.612
Current smoker	5 (4.6)	13 (20.6)	7 (16.7)	<0.001	0.014	0.612
Smoking, pack-years	0 (0–6)	55 (40–68)	40 (29–54)	<0.001	<0.001	0.280
***Comorbidities***						
Rhinitis	47 (43.1)	4 (6.3)	13 (31.0)	<0.001	0.171	0.001
Atopic dermatitis	9 (8.3)	0 (0.0)	5 (11.9)	0.019	0.489	0.005
Sinusitis	22 (20.2)	3 (4.8)	9 (21.4)	0.006	0.865	0.009
Hypertension	42 (38.5)	27 (42.9)	20 (47.6)	0.577	0.309	0.631
Hyperlipidemia	21 (19.3)	10 (15.9)	14 (33.3)	0.577	0.066	0.037
Diabetes mellitus	18 (16.5)	7 (11.1)	3 (7.1)	0.225	0.092	0.497
***Laboratory data***						
WBC, /µL	6200 (5050–7450)	6000 (5100–7000)	6850 (5900–7530)	>0.999	0.150	0.098
Blood neutrophil count, /µL	3550 (2840–4830)	3760 (3130–4650)	3820 (3260–4940)	>0.999	0.494	>0.999
Blood eosinophil count, /µL	180 (90–340)	140 (100–200)	360 (190–530)	0.161	0.006	<0.001
Blood eosinophil count, %	2.8 (1.4–5.6)	2.4 (1.6–3.4)	4.3 (2.6–8.3)	0.103	0.053	<0.001
d–ROMs, CARR U	335 (304–388)	358 (334–402)	370 (344–428)	0.018	0.004	>0.999
CRP, mg/dL	0.08 (0.04–0.19)	0.14 (0.05–0.35)	0.14 (0.04–0.31)	0.196	0.085	>0.999
SAA, µg/mL	5.1 (3.0–10.8)	6.1 (0.0–12.0)	6.1 (3.2–11.6)	>0.999	>0.999	>0.999
IL-6, pg/mL	1.8 (1.2–3.5)	3.1 (1.7–4.7)	3.7 (2.1–6.9)	0.003	<0.001	0.199
IL-8, pg/mL	15.7 (11.3–21.4)	21.0 (16.0–31.9)	19.2 (14.3–24.9)	<0.001	0.063	0.736
TNF-α, pg/mL	0.7 (0.3–1.0)	0.7 (0.5–1.0)	0.9 (0.6–1.3)	0.286	0.003	0.283
IgE, IU/mL	110 (40–340)	40 (20–150)	150 (50–640)	0.002	0.608	<0.001
specific IgE against antigens, number	3 (1–6)	1 (0–2)	3 (1–7)	<0.001	>0.999	0.002
***Pulmonary function test***						
FVC, % predicted	98.3 (86.5–107.3)	96.4 (81.0–111.4)	95.9 (86.3–104.9)	>0.999	>0.999	>0.999
FEV_1_, % predicted	92.2 (82.9–101.7)	74.1 (50.5–87.0)	71.1 (58.1–80.0)	<0.001	<0.001	0.820
FEV_1_/FVC, %	75.2 (68.4–82.1)	60.1 (46.1–67.1)	56.3 (48.4–64.1)	<0.001	<0.001	>0.999
FeNO, ppb	26 (17–45)	21 (13–28)	38 (24–59)	0.018	0.062	<0.001
***Symptom***						
ACT Score	22 (19–24)	NA	20 (18–23)	NA	0.052	NA
CAT Score	NA	10 (5–19)	13 (9–17)	NA	NA	0.224
SGRQ Score	20.2 (8.1–33.2)	26.1 (14.0–39.8)	28.8 (17.0–41.4)	0.225	0.075	>0.999
***Treatment***						
ICS use	102 (93.6)	16 (25.4)	34 (81.0)	<0.001	0.020	<0.001
LABA use	82 (75.2)	49 (77.8)	36 (85.7)	0.379	0.162	0.310
LAMA use	23 (21.1)	46 (73.0)	27 (64.3)	<0.001	<0.001	0.341
OCS use	14 (12.8)	0 (0.0)	4 (9.5)	0.003	0.573	0.012
Biologics use	14 (12.8)	0 (0.0)	3 (7.1)	0.003	0.321	0.031

Data presented are median (interquartile range) or number (%). BA, bronchial asthma; COPD, chronic obstructive pulmonary disease; ACO, asthma–COPD overlap; WBC, white blood cells; d–ROMs, derivatives–eactive oxygen metabolites; CRP, C-reactive protein; SAA, serum amyloid A; IL, interleukin; TNF-α, tumor necrosis factor alpha; IgE, immunoglobulin E; FVC, forced vital capacity; FEV_1_, forced expiratory volume in 1 s; FeNO, fractional exhaled nitric oxide; ACT, asthma control test; CAT, COPD assessment test; SGRQ, St. George’s Respiratory Questionnaire; ICS, inhaled corticosteroids; LABA, long-acting beta2-agonists; LAMA, long-acting muscarinic antagonists; OCS, oral corticosteroids; NA, not available.

**Table 3:** Correlation between serum d–ROMs levels and biomarkers or questionnaires in BA, COPD and ACO.

	BA		COPD		ACO	
	*rs*	*p*	*rs*	*p*	*rs*	*p*
Age	0.275	0.004	0.028	0.829	0.241	0.124
pack-years	−0.171	0.076	−0.057	0.659	−0.076	0.633
WBC	0.253	0.008	0.265	0.036	0.023	0.884
Neutrophils	0.293	0.002	0.265	0.036	0.257	0.100
Eosinophils	−0.123	0.204	0.431	<0.001	−0.003	0.835
IgE	0.061	0.526	−0.020	0.879	0.145	0.360
CRP	0.375	<0.001	0.431	<0.001	0.440	0.004
SAA	0.316	0.001	0.408	0.001	0.487	0.001
IL-6	0.270	0.004	0.487	<0.001	0.483	0.001
IL-8	0.232	0.015	0.118	0.358	0147	0.352
TNF-α	−0.038	0.691	0.035	0.787	0.417	0.006
FeNO	0.011	0.912	−0.160	0.212	−0.039	0.808
ACT Score	−0.388	<0.001	NA	NA	−0.280	0.072
CAT Score	NA	NA	0.442	<0.001	0.329	0.034
SGRQ Score	0.391	<0.001	0.406	0.001	0.440	0.004

d–ROMs, derivatives–reactive oxygen metabolites; BA, bronchial asthma; COPD, chronic obstructive pulmonary disease; ACO, asthma–COPD overlap; WBC, white blood cells; IgE, immunoglobulin E; CRP, C-reactive protein; SAA, serum amyloid A; IL, interleukin; TNF-α, tumor necrosis factor alpha; FeNO, fractional exhaled nitric oxide; ACT, asthma control test; CAT, COPD assessment test; SGRQ, St. George’s Respiratory Questionnaire; NA, not available.

## Data Availability

The data that support the findings of this study are available from the corresponding authors on reasonable request. Before this request, users should obtain permission from the local ethics committee.

## Abbreviations

ACO	asthma–COPD overlap
ACT	asthma control test
ATS	American Thoracic Society
BA	bronchial asthma
CAT	COPD assessment test
COPD	chronic obstructive pulmonary disease
CRP	C-reactive protein
D_LCO_	diffusing capacity of the lung for carbon monoxide
d–ROMs	derivatives–reactive oxygen metabolites
ERS	European Respiratory Society
FeNO	fractional exhaled nitric oxide
FEV_1_	forced expiratory volume in 1 s
FVC	forced vital capacity
GINA	Global Initiative for Asthma
GOLD	Global Initiative for Chronic Obstructive Lung Disease
HRCT	high-resolution computed tomography
ICS	inhaled corticosteroids
IgE	immunoglobulin E
IL	interleukin
JRS	Japanese Respiratory Society
LAA	low attenuation area
LAMA	long-acting muscarinic antagonists
LABA	long-acting beta2-agonists
OCS	oral corticosteroids
ROC	receiver operating characteristic
ROS	reactive oxygen species
SAA	serum amyloid A
SGRQ	St. George’s Respiratory Questionnaire
TNF-α	tumor necrosis factor alpha
WBC	white blood cell
